# The lung allocation score could evaluate allocation systems in countries that do not use the score

**DOI:** 10.1371/journal.pone.0214853

**Published:** 2019-04-03

**Authors:** Woo Sik Yu, Jee Won Suh, Seung Hwan Song, Hyo Chae Paik, Song Yee Kim, Moo Suk Park, Jin Gu Lee

**Affiliations:** 1 Department of Thoracic and Cardiovascular Surgery, Ajou University School of Medicine, Suwon, Republic of Korea; 2 Department of Thoracic and Cardiovascular Surgery, College of Medicine, Yonsei University, Seoul, Republic of Korea; 3 Division of Pulmonary and Critical Care Medicine, Department of Internal Medicine, Yonsei University College of Medicine, Yonsei University Health System, Seoul, South Korea; Fordham University, UNITED STATES

## Abstract

**Background:**

Evaluating allocation system effects on lung transplantation and determining systemic flaws is difficult. The purpose of this study was to assess the Korean urgency-based lung allocation system using the lung allocation score.

**Methods:**

We reviewed transplantation patients retrospectively. Candidates were classified into groups based on urgency. Status 0 designated hospitalized patients requiring ventilator and/or extracorporeal life support. The lung allocation score was calculated based on the recipient’s condition at transplantation.

**Results:**

One-hundred-twenty-three Status 0, 1, and 2/3 patients (40, 71, and 12, respectively) were enrolled. The median waiting time was 68 days. Nineteen Status 0 patients who received lung transplants deteriorated from non-Status 0 (median, 64 days). The lung allocation score showed a bimodal distribution (peaks around 45 and 90, corresponding with non-Status 0 and Status 0, respectively). Status 0 and the lung allocation score were independent risk factors for poor survival after adjustment for confounders (Status 0, hazard ratio, 2.788, *p* = 0.001; lung allocation score, hazard ratio, 1.025, *p* < 0.001). The lung allocation score cut-off for survival was 44. On dividing the non-Status 0 patients into 2 groups using the cut-off values and regrouping into Status 0, non-Status 0 with high lung allocation score (> 44), and non-Status 0 with low lung allocation score (< 44), we observed that non-Status 0 with high lung allocation score patients had better survival than Status 0 patients (*p* = 0.020) and poorer survival than non-Status 0 with low lung allocation score patients (*p* = 0.018).

**Conclusions:**

The LAS demonstrated the characteristics of LTx recipients in Korea and the Korean allocation system needs to be revised to reduce the number of patients receiving LTx in Status 0. The LAS system could be used as a tool to evaluate lung allocation systems in countries that do not use the LAS system.

## Introduction

Lung transplantation (LTx) has been a standard treatment for end-stage lung disease [1]. Some LTx candidates die on the waiting list before they undergo LTx because of a donor shortage. Health authorities must have an allocation system for donor lungs. Allocation systems have been designed empirically and differ between countries; they are based on urgency, waiting time, benefits, or a combination of these criteria in most countries [2, 3]. These allocation systems can affect the characteristics of patients who undergo LTx and the outcomes in each country. However, it is difficult to evaluate the effects of allocation systems on LTx, determine systemic flaws, and revise the systems, especially in low-volume countries.

In Korea, a large portion of LTxs have been performed on patients with very urgent conditions, and this may worsen LTx outcomes. Among LTx recipients, 34.1–63.4% of patients were receiving preoperative mechanical ventilation and 19.5–46.3% of patients were supported by extracorporeal membrane oxygenation (ECMO) [4–6]. This may be due to the urgency-based allocation system, which assigns the highest priority to patients supported by mechanical ventilation or ECMO.

In 2005, the lung allocation score (LAS) system was introduced [7]. This system analyzed the UNOS national database by using a Cox proportional hazard model. With this model, the urgency measure (1-year survival without a transplant) and survival measure (1-year survival with a transplant) are predicted. The LAS is calculated as the net transplant benefit measure (survival measure–urgency measure) minus the urgency measure and normalized to a 0 to 100 scale. Therefore, the LAS can be analyzed as a continuous variable.

We hypothesized that the LAS could be used as a tool to evaluate allocation systems. The LTx program at our institution has the largest volume in Korea; nearly half of Korean LTxs have been conducted here [6]. This study aimed to analyze the characteristics of the urgency-based allocation system and LTx outcomes in Korea by simulating the LAS for LTx recipients at our institution.

## Materials and methods

### Patients

From January 2012 to December 2016, all patients who underwent LTx at Yonsei University College of Medicine were included except for patients who received combined solid organ transplants. The follow-up ended in July 2017. This retrospective study was approved by the Institutional Review Board of Severance Hospital (IRB No. 4-2018-0442). The requirement for informed consent was waived.

All organs were recovered en bloc from mechanically assisted brain-dead donors. Clamshell incision of the fourth intercostal space has been the preferred surgical approach in double LTx. Double LTx was performed sequentially. The right side was usually implanted first. All patients received standard triple immunosuppression with a calcineurin inhibitor (cyclosporine or tacrolimus), mycophenolate mofetil, and methylprednisolone.

### The donor lung allocation system in Korea and calculation of the LAS

Urgency status definitions are described in Table 1 [4]. Donor lungs are allocated to the most urgent patients based on urgency status. Among patients with the same status, candidates are selected using a scoring system with waiting time, blood type, presence of infectious disease, distance between the donor and recipient hospitals, age, primary lung diagnosis, and difference in the donor’s and recipient’s estimated lung volumes. Patients’ urgency statuses at the time of being placed on the list and LTx were collected. Waiting time was measured from the date of enrollment for LTx to the date of the operation. The LAS, urgency measure, and survival measure were calculated retrospectively based on the patient’s condition at the time of LTx evaluation in July 2017 [8].

**Table 1:** Definition of urgency status in Korea.

Urgency Status	Indications
Status 0	Patients are connected to a ventilator or an extracorporeal membrane oxygenation
Status 1	One of the following
	NYHA stage IV patients with a PaO_2_ < 55 mmHg in room air
	NYHA stage IV patients with an average pulmonary pressure > 65 mmHg or an average right atrial pressure > 15 mmHg
	Cardiac index < 2 L/min/m^2^
Status 2	One of the following
	Forced expiratory volume in 1 second < 25% on the pulmonary function test
	PaO_2_ < 60 mmHg in room air
	Average right atrial pressure: 10–15 mmHg
	Average pulmonary pressure: 55–65 mmHg
	Cardiac index: 2–2.5 L/min/m^2^
Status 3	Patients without any of the above conditions but requiring a lung transplant

### Data collection

Clinical data were collected from electronic medical records. Diagnoses were divided into three groups. Restrictive diseases included idiopathic pulmonary fibrosis, connective tissue disease-related interstitial lung disease, other interstitial lung disease, graft-versus-host disease after hematopoietic stem cell transplantation, acute respiratory distress syndrome, and re-transplant. Obstructive diseases included chronic obstructive pulmonary disease, bronchiectasis, and lymphangioleiomyomatosis. Pulmonary vascular disease included primary pulmonary hypertension. The most recent pulmonary function test, 6-minute walking distance (6MWD), laboratory results, and medical condition were analyzed. Pulmonary hypertension was defined as a mean pulmonary arterial pressure ≥ 25 mmHg by right heart catheterization or a systolic pulmonary arterial pressure ≥ 40 mmHg by echocardiography. Survival was measured from the date of LTx to the date of death.

### Statistical analysis

The statistical analyses were performed using SPSS version 23.0 (IBM Corp, USA) and R version 3.4.4 (R Foundation for Statistical Computing, Vienna, Austria). Clinical variables are described as the mean ± standard deviation or median (interquartile range [IQR]) for continuous variables and as the frequency (percentage) for categorical variables. Continuous variables were compared using a one-way analysis of variance with a post hoc Bonferroni correction or the Mann-Whitney *U* test with a post hoc test using the Tukey–Kramer method. Categorical variables were compared using the chi-squared test or Fisher’s exact test. The correlations between the LAS, urgency measure, and survival measure were calculated using the Pearson correlation test. The survival analysis was performed using the Kaplan-Meier method and comparisons were made using the log-rank test; the p-value was adjusted for multiple comparisons [9]. A Cox proportional hazards analysis was performed for the multivariate analysis. Maximally selected rank statistics were used to detect the optimal LAS cut-off for waiting list mortality [10]. The cut-off that best separated patient outcomes according to a maximum relative risk and a minimum *p*-value were chosen; this *p*-value was adjusted to account for multiple comparisons [11]. *P*-values less than 0.05 were considered statistically significant.

## Results

**Fig 1:**
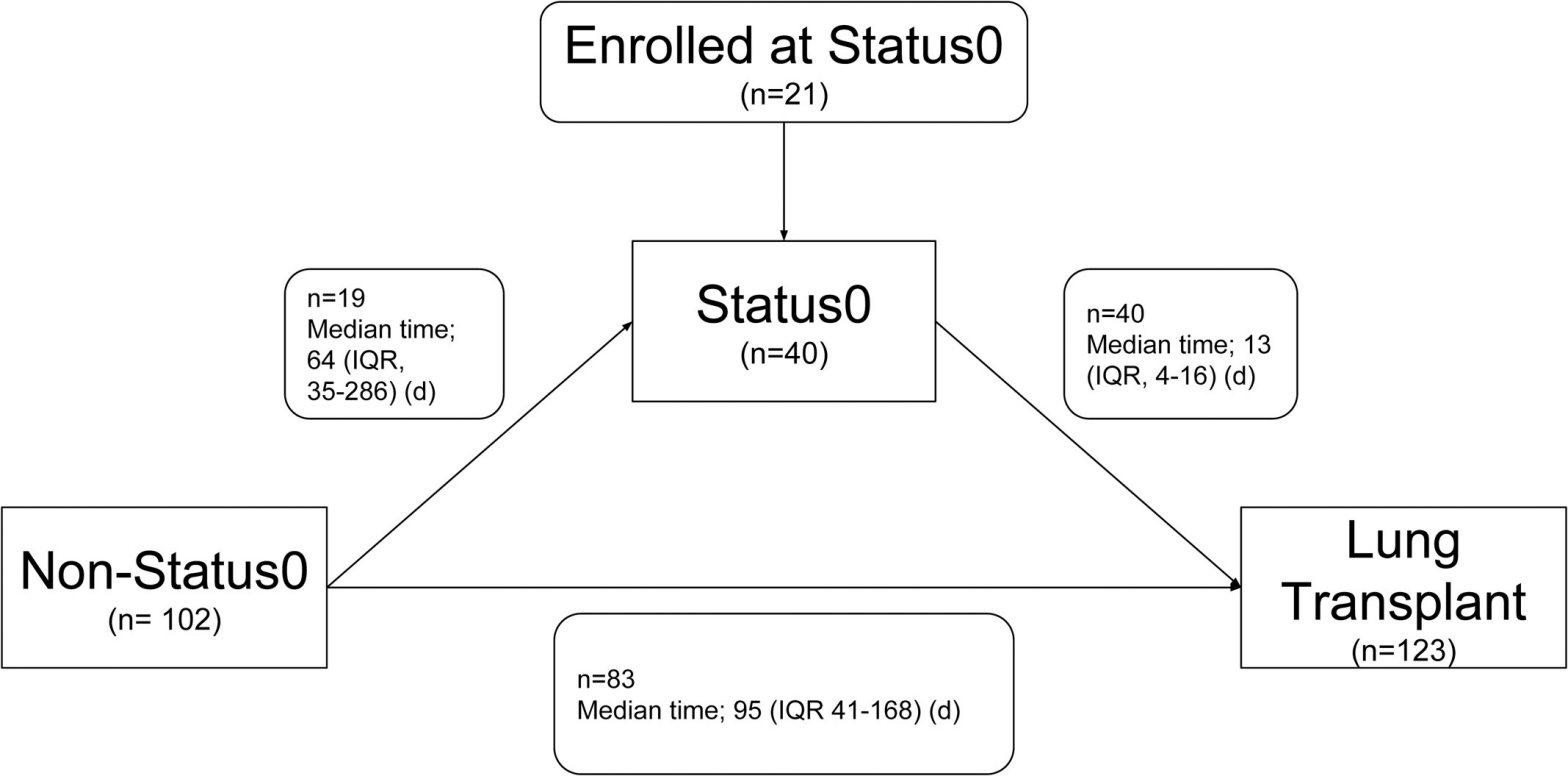
Changes in Korean urgency status during the waiting period. Abbreviation: IQR, interquartile range.

During the study period, a total of 125 LTxs were performed. Except for two patients who received combined transplants (one liver and one kidney), 123 patients were included in the study. Among all patients, the median waiting time was 68 (IQR, 117) days. Among 102 non-Status 0 patients (Status 1 or Status 2/3) at the time of enrollment for LTx, 19 (18.6%) patients deteriorated to Status 0 in a median of 64 (IQR, 251) days. Forty Status 0 patients received LTx in a median of 13 (IQR, 12) days (Fig 1).

Patient characteristics at the time of transplantation are summarized in Table 2. Sixty-six (53.7%) patients received LTx for idiopathic pulmonary fibrosis; it was the most frequent indication for LTx among all urgency statuses. Status 0 patients had a lower 6MWD, higher total bilirubin, shorter waiting time, and lower donor partial pressure of oxygen in the arterial blood/fraction of inspired oxygen ratio than Status 1 (*p* = 0.001, *p* = 0.053, *p* = 0.005, and *p* = 0.003, respectively) or Status 2/3 patients (*p* < 0.001, *p* = 0.386, *p* = 0.015, and *p* = 0.041, respectively). Between Status 1 and Status 2/3 patients, only the 6MWD showed a significant difference (*p* = 0.018). Among Status 0 patients, 36 (90.0%) and 22 (55.0%) patients were dependent on mechanical ventilation and ECMO, respectively.

**Table 2:** Baseline patient characteristics at the time of transplantation.

	Total (n = 123)	Status 0 (n = 40)	Status 1 (n = 71)	Status 2/3 (n = 12)	*p*-value
Age (y)	52.16 ± 13.07	52.20 ± 13.85	51.20 ± 12.71	57.75 ± 12.13	0.277
Male (%)	71 (57.7)	23 (57.5)	41 (57.7)	7 (58.3)	> 0.999
BMI (kg/m^2^)	20.55 ± 3.56	19.91 ± 3.30	21.17 ± 3.66	19.01 ± 3.17	0.058
Diabetes (%)	21 (17.1)	4 (10.0)	15 (21.1)	2 (16.7)	0.388
Primary diagnosis					0.009
IPF (%)	66 (53.7)	21 (52.5)	37 (52.1)	8 (66.7)	
CTD-related ILD (%)	16 (13.0)	1 (2.7)	15 (21.1)	0 (0)	
Other ILD (%)	6 (4.9)	4 (10.0)	2 (2.8)	0 (0)	
GVHD (%)	12 (9.8)	6 (15.0)	6 (8.5)	0 (0)	
COPD (%)	2 (1.6)	1 (2.5)	0 (0)	1 (8.3)	
Bronchiectasis (%)	9 (7.3)	2 (5.0)	6 (8.5)	1 (8.3)	
PPH (%)	2 (1.6)	1 (2.5)	1 (1.4)	0 (0)	
LAM (%)	4 (3.3)	0 (0)	2 (2.8)	2 (16.7)	
ARDS (%)	3 (2.4)	3 (7.5)	0 (0)	0 (0)	
Eosinophilic granuloma (%)	1 (0.8)	0 (0)	1 (1.4)	0 (0)	
Re-transplant (%)	2 (1.6)	1 (2.5)	1 (1.4)	0 (0)	
Diagnostic group^a^					0.173
Restrictive (%)	106 (86.2)	36 (90.0)	62 (87.3)	8 (66.7)	
Obstructive (%)	15 (12.2)	3 (7.5)	8 (11.3)	4 (33.3)	
Pulmonary vascular (%)	2 (1.6)	1 (2.5)	1 (1.4)	0 (0)	
FVC (% predicted)^b^	42.12 ± 16.71	40.66 ± 16.51	41.94 ± 15.61	46.67 ± 23.32	0.576
FEV1 (% predicted)^b^	44.39 ± 20.31	45.23 ± 22.46	44.59 ± 18.59	41.16 ± 25.78	0.838
6MWD (m)	159 ± 159	76 ± 134	183 ± 135	315 ± 212	<0.001
Pulmonary hypertension (%)	74 (60.2)	23 (57.5)	43 (60.6)	8 (66.7)	0.900
Mean PAP^c^ (%)	28.24 ± 10.34	28.69 ± 14.95	28.27 ± 9.68	27.50 ± 7.33	0.963
Laboratory values					
pCO_2_^d^	43.39 ± 14.92	46.88 ± 15.92	40.97 ± 12.63	45.48 ± 21.29	0.121
Creatinine	0.61 ± 0.23	0.54 ± 0.33	0.63 ± 0.15	0.67 ± 0.22	0.102
Total bilirubin	0.76 ± 1.3	1.17 ± 2.19	0.56 ± 0.31	0.53 ± 0.33	0.048
Medical conditions					
ICU (%)	40 (32.5)	37 (92.5)	3 (4.2)	0 (0)	< 0.001
GW (%)	20 (16.3)	3 (7.5)	17 (23.9)	0 (0)	0.026
Not hospitalized (%)	63 (51.2)	0 (0)	51 (71.8)	12 (100)	< 0.001
Vasopressor use (%)	27 (22.0)	25 (62.5)	2 (2.8)	0 (0)	<0.001
High-flow oxygen therapy (%)	10 (8.1)	1 (2.5)	9 (12.7)	0 (0)	0.143
Mechanical ventilation (%)	36 (29.3)	36 (90.0)	0 (0)	0 (0)	< 0.001
ECMO (%)	22 (17.9)	22 (55.0)	0 (0)	0 (0)	< 0.001
Double-lung transplant (%)	113 (91.9)	36 (90.0)	67 (94.4)	10 (83.3)	0.265
Waiting time (d)	68 (19–136)	23.5 (5.2–81.7)	84 (39–148)	134 (61.2–196.5)	0.002
Donor characteristics					
Age (y)	41.44 ± 11.77	39.45 ± 13.26	42.41 ± 11.11	42.33 ± 10.29	0.432
Ventilator duration (h)	148 ± 106	158 ± 92	145 ± 117	133 ± 79	0.721
P/F ratio	437 ± 95	395 ± 107	455 ± 76	470 ± 112	0.002
Ischemic duration (m)	239 ± 83	250 ± 86	239 ± 83	203 ± 65	0.233

^a^ Restrictive diseases included IPF, CTD-ILD, other ILD, GVHD after HSCT, ARDS, and re-transplant. Obstructive diseases included COPD, bronchiectasis, and LAM. Pulmonary vascular diseases included PPH

^b^ not available for 11 Status 0 patients

^c^ Available for 78 patients (Status 0, 12; Status 1, 56; Status 2/3, 10)

^d^ not available for three Status 1 patients.

Abbreviations: 6MWD, 6-minute walking distance; ARDS, acute respiratory distress syndrome; BMI, body mass index; COPD, chronic obstructive pulmonary disease; CTD-ILD, connective tissue disease-related interstitial lung disease; ECMO, extracorporeal membrane oxygenation; FEV1, forced expiratory volume in 1 second; FVC, forced vital capacity; GVHD, graft versus host disease; GW, general ward; HSCT, hematopoietic stem cell transplantation; ICU, intensive care unit; ILD, interstitial lung disease; IPF, idiopathic pulmonary fibrosis; LAM, lymphangioleiomyomatosis; PAP, pulmonary artery pressure; P/F ratio, partial pressure of oxygen in the arterial blood/fraction of inspired oxygen ratio; PPH, primary pulmonary hypertension

**Fig 2:**
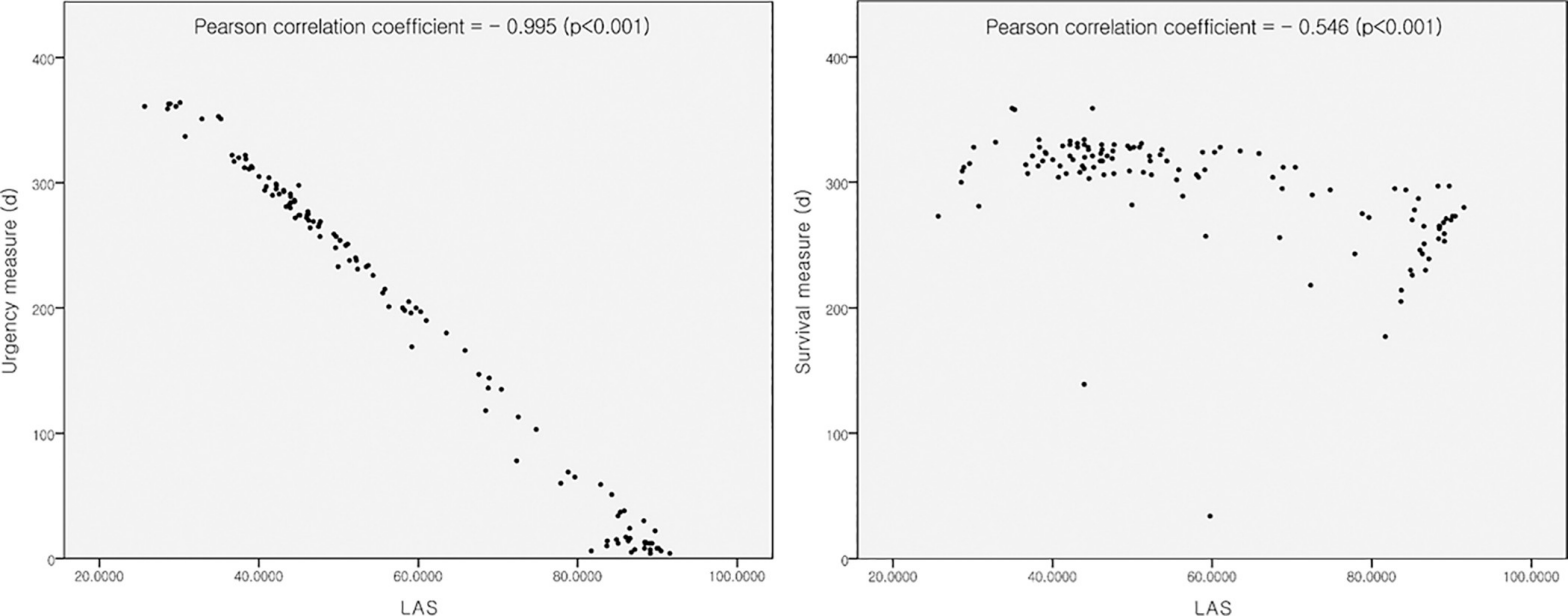
Correlations between the lung allocation score, urgency measure, and survival measure. Abbreviation: LAS, lung allocation score.

**Fig 3:**
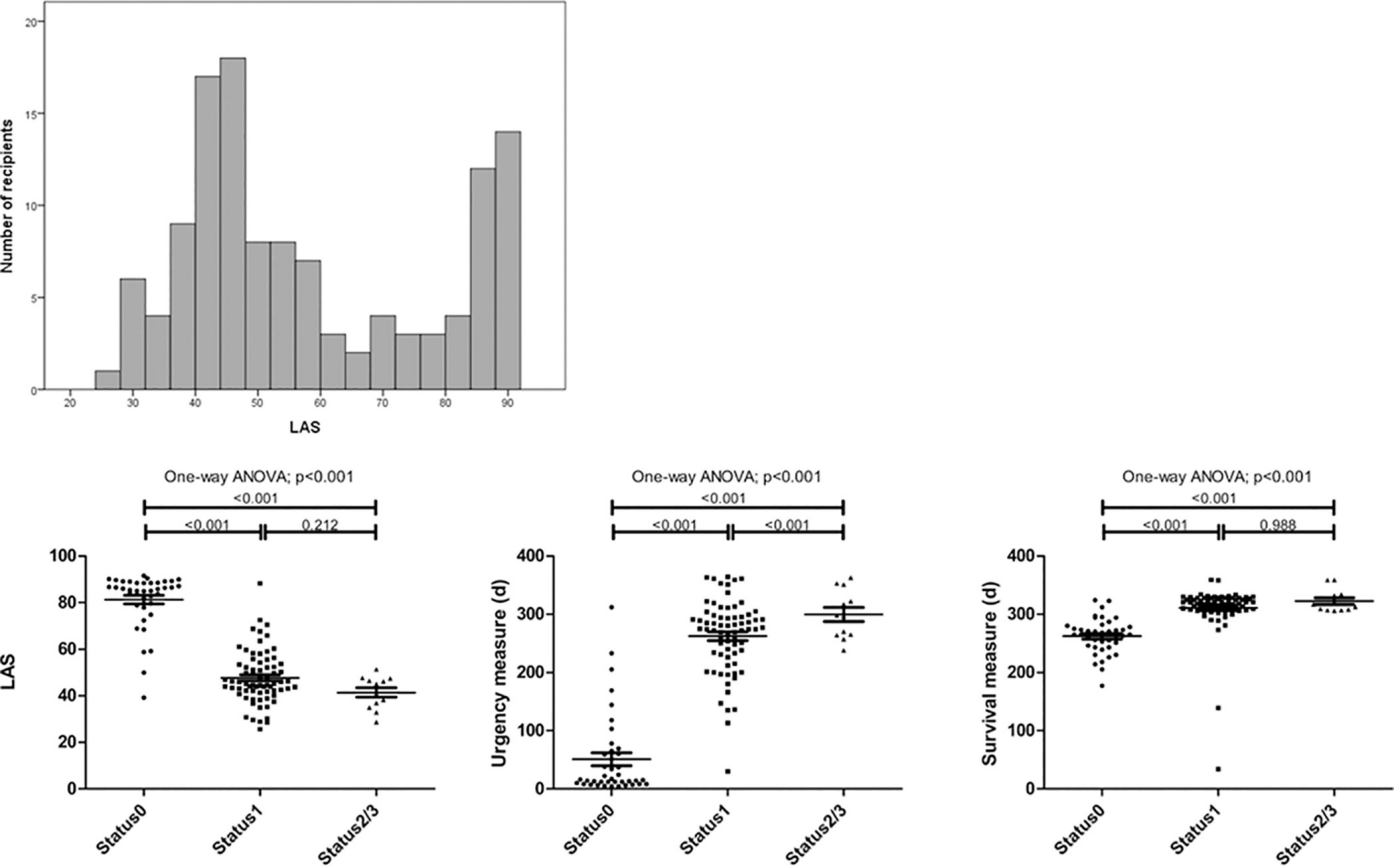
Distributions of lung allocation scores among patients who received lung transplants. Abbreviations: ANOVA, analysis of variance; LAS, lung allocation score.

Although the LAS showed negative correlations with both urgency and survival measures, it seemed more correlated with the urgency measure (Pearson correlation coefficient: LAS and urgency measure, -0.995, *p* < 0.001 versus LAS and survival measure, -0.546, *p* < 0.001) (Fig 2). The LAS in our patients showed a bimodal distribution with peaks around 45 and 90; the latter peak corresponded with Status 0 (Fig 2). Status 0 patients had much higher LAS values compared with Status 1 and Status 2/3 patients (Status 0, 81.2 ± 11.7; Status 1, 47 ± 11.3; Status 2/3, 41.3 ± 7.0) (Fig 3).

**Fig 4:**
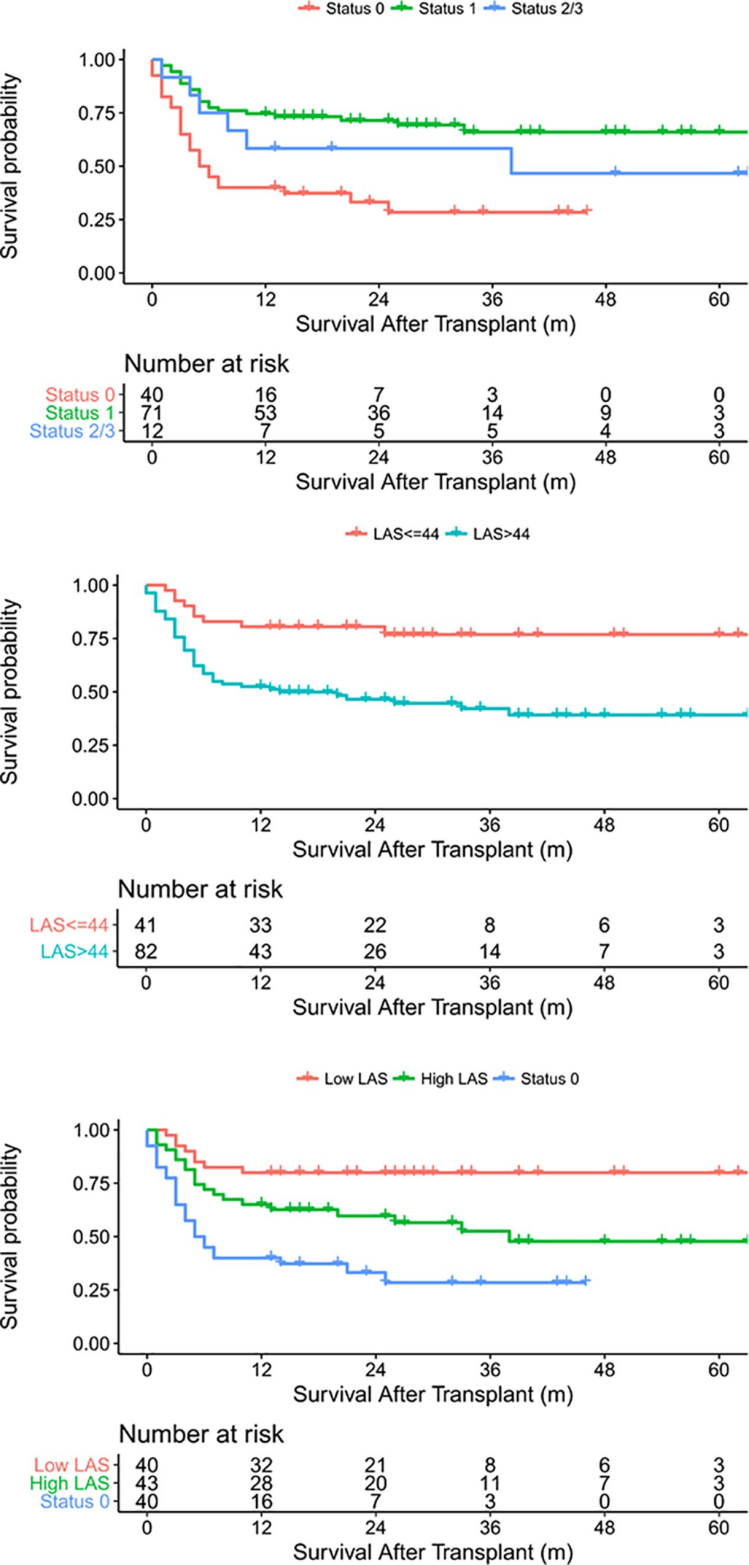
Survival according to urgency status and the lung allocation score. Abbreviation: LAS, lung allocation score.

The mean follow-up duration after LTx was 20.5 ± 17.6. Status 0 and the LAS were significant prognostic factors in the multivariate Cox proportional hazard analysis after adjusting for age, sex, diagnostic group, and donor characteristics (age, sex, ventilation duration, partial pressure of oxygen in the arterial blood/fraction of inspired oxygen ratio, and ischemic duration) (Table 3). Status 0 patients had lower survival than Status 1 patients (*p* < 0.001); however, there was no significant difference between Status 1 and Status 2/3 (*p* = 0.290). By maximally selected rank statistics, the optimal LAS cut-off value was 44. Patients with a LAS greater than 44 had poorer survival than patients with a LAS less than 44 (*p* < 0.001). On dividing non-Status 0 patients into 2 groups using the cut-off value and regrouping the patients as Status 0, non-Status 0 with high LAS (LAS > 44), and non-Status 0 with low LAS (LAS < 44), we observed that non-Status 0 with high LAS patients had better survival than Status 0 patients (*p* = 0.020) and poorer survival than non-Status 0 with low LAS patients (*p* = 0.018) (Fig 4).

**Table 3:** Univariate and multivariate hazard ratios of death based on Korean urgency status and the lung allocation score.

	HR	95% CI	*p*-value
Status 0			
Unadjusted	2.905	1.703–4.955	< 0.001
Age-adjusted	2.700	1.579–4.617	< 0.001
Multivariate	2.730	1.592–4.680	< 0.001
Multivariate + donor	2.788	1.560–4.980	0.001
LAS			
Unadjusted	1.026	1.012–1.039	< 0.001
Age-adjusted	1.025	1.011–1.039	< 0.001
Multivariate	1.025	1.011–1.039	< 0.001
Multivariate + donor	1.025	1.010–1.040	0.001

Multivariate values are adjusted for age, sex, and diagnostic group. Donor characteristics are adjusted for age, sex, hours of ventilation, partial pressure of oxygen in the arterial blood/fraction of inspired oxygen ratio, and ischemic duration. Abbreviations: CI, confidence interval; HR, hazard ratio; LAS, lung allocation score

## Discussion

The lung allocation system affects the entire LTx process including waiting list mortality and post-transplant survival. Sufficient time and cases are needed to evaluate the effects of lung allocation systems [12–15]. However, outside North America and Europe, where there are large LTx volumes, it is difficult for countries to have enough data to evaluate their allocation system, find flaws, and revise the system. Therefore, studies regarding the effects of lung allocation systems have rarely been reported outside North America and European countries.

The LAS was introduced in the United States in 2005; it was adopted in Germany in 2011 and in the Netherlands in 2014. More than 60% of lungs are allocated using the LAS worldwide [2]. The LAS is represented as a continuous variable on a 0 to 100 scale. It is possible to rank LTx candidates and compare them between countries, at least indirectly. Although, the urgency measure and post-transplant survival measure are used to calculate the LAS, urgency measure is double-weighted compared with the post-transplant survival measure. In our study, the LAS seemed more correlated with the urgency measure than the post-transplant survival measure (Pearson correlation coefficient: LAS and urgency measure, -0.995, *p* < 0.001 versus LAS and survival measure, -0.546, *p* < 0.001). Using these LAS characteristics, we evaluated the Korean lung allocation system and Korean lung allocation characteristics.

In our cohort, the LAS showed a bimodal distribution with peaks around 45 and 90. The peak around 90 correlated with Status 0 and the peak around 45 correlated with Status 1 and Status 2/3. The difference in the LAS between Status 0 and Status 1 was quite large. On the other hand, there was no significant difference between Status 1 and Status 2/3 in the LAS. Because donor lungs are allocated considering various factors including waiting time in the same urgent status, our allocation system is quite similar to the high-emergency lung transplantation system (HELTx), which is used in some European countries such as France [2, 3]. The HELTx gives priority to candidates with conditions posing an immediate threat to life while the remainder receive donor organs based on the waiting time, which is similar to status 0 in the Korean lung allocation system [16]. Riou et al. recently published a study simulating the HELTx with an agent-based model of LTx waiting queues using *NetLogo* [3]. In this study, when donor organs were available for 96% of LTx candidates, the percentage of patients on the high-priority list who received LTx increased from 4.0% to 9.1% between the first and fifteenth years of the simulation. However, when donor organs were available for 80% of LTx candidates, it increased from 11.6% to 32.5%. This simulation may explain the allocation in Korea. The HELTx-like allocation system and shortage of donor lungs in Korea may have resulted in 32.5% of patients who received LTx to be Status 0. Moreover, non-Status 0 patients with a higher LAS cannot have priority before deterioration to Status 0 compared with other non-Status 0 patients. In our study, 18.6% of non-Status 0 patients at the time of LTx enrollment deteriorated to Status 0.

Patients with a higher LAS in the LAS allocation system and high-priority patients in the HELTx system may have decreased survival following LTx [16–18]. In this study, Status 0 and the LAS were independent prognostic factors for survival after adjusting for age, sex, diagnosis, and donor characteristics. When patients were regrouped as Status 0, high LAS, and low LAS using a cut-off value of 44, non-Status 0 with high LAS patients had better survival than Status 0 patients. This suggests that when these patients have priority over low LAS patients, they can receive LTx before they deteriorate to Status 0. Thus, LTx outcomes might be improved if fewer patients receive LTx in Status 0. This finding may help to revise the allocation system in Korea. Because the LAS mainly ranks LTx candidates according to urgency rather than categorization, introduction of the LAS system could decrease the LAS of LTx recipients in Korea. Schuba et al. reported a 5-year experience with the LAS at a single German LTx center in which the LAS values of patients receiving LTx decreased slightly over time from 50.6 ± 18.0 in the first year to 45.2 ± 16.2 in the fifth year [15].

Our study has several limitations. First, it has a retrospective design. The LAS was calculated retrospectively using medical records. Our LAS results cannot be compared directly with study results from countries using the LAS for allocation. Second, although, we showed the necessity of revising the lung allocation system in Korea, we could not suggest definite changes or criteria. Even if the LAS is introduced in Korea, we cannot know how the LAS, which is based on data from the United States, would predict medical urgency and post-transplant survival. Third, this study was conducted at a single center in Korea; however, we performed nearly half of the LTx cases in Korea. Other LTx centers in Korea could have different LTx candidate selection strategies, which may affect the characteristics of LTx recipients and outcomes [4, 5].

In conclusion, we have shown that the LAS demonstrated the characteristics of LTx recipients in Korea and that the Korean allocation system needs to be revised to reduce the number of patients receiving LTx in Status 0. Apart from donor lung allocation, the LAS system could be used as a tool to evaluate lung allocation systems in countries that do not use the LAS system.

## Supporting information

S1 Dataset Original data for study subjects. (XLSX)
